# Expression of Growth Hormone-Releasing Hormone and Its Receptor Splice Variants in a Cohort of Hungarian Pediatric Patients with Hematological and Oncological Disorders: A Pilot Study

**DOI:** 10.3390/ijms25168831

**Published:** 2024-08-14

**Authors:** Éva Juhász, Zsuzsanna Szabó, Andrew V. Schally, József Király, Petra Fodor, Gábor Kónya, Balázs Dezső, Erzsébet Szabó, Gábor Halmos, Csongor Kiss

**Affiliations:** 1 Department of Pediatrics, Faculty of Medicine, University of Debrecen, 4032 Debrecen, Hungary; juhasze@med.unideb.hu; 2 Department of Biopharmacy, Faculty of Pharmacy, University of Debrecen, 4032 Debrecen, Hungary; szabo.zsuzsanna@pharm.unideb.hu (Z.S.); kiraly.jozsef@pharm.unideb.hu (J.K.); fodor.petra@pharm.unideb.hu (P.F.); konya.gabor@pharm.unideb.hu (G.K.); 3 Veterans Affairs Medical Center, Endocrine, Polypeptide and Cancer Institute, Miami, FL 33125, USA; aschally@med.miami.edu; 4 Department of Pathology, Miller School of Medicine, University of Miami, Miami, FL 33101, USA; 5 Department of Medicine, Divisions of Hematology-Oncology and Endocrinology, Miller School of Medicine, University of Miami, Miami, FL 33101, USA; 6 Sylvester Comprehensive Cancer Center, University of Miami, Miami, FL 33136, USA; 7 Department of Pathology, Faculty of Medicine, University of Debrecen, 4032 Debrecen, Hungary; bdezso@med.unideb.hu; 8 Department of Pharmacology, Faculty of Pharmacy, HUN-REN-DE Pharmamodul Research Group, University of Debrecen, Nagyerdei krt. 98, 4032 Debrecen, Hungary; erzsebet.szabo@med.unideb.hu

**Keywords:** hematological-oncological disorders in children, GHRH, GHRH receptor, splice variant

## Abstract

Hematological and oncological diseases are still among the leading causes of childhood mortality. Expression of growth hormone-releasing hormone (GHRH) and its receptors (GHRH-R) has been previously demonstrated in various human tumors, but very limited findings are available about the presence and potential function of GHRH-Rs in oncological and hematological disorders of children. In this study, we aimed to investigate the expression of mRNA for GHRH and splice variant 1 (SV) of GHRH-R in 15 pediatric hematological/oncological specimens by RT-PCR. The presence and binding characteristics of GHRH-R protein were also studied by Western blot and ligand competition assays. Of the fifteen specimens studied, eleven pediatric samples (73%) showed the expression of mRNA for GHRH. These eleven samples also expressed mRNA for GHRH receptor SV1. GHRH-R protein was found to be expressed in two benign tumor samples and five malignant tumors examined by Western blot. The presence of specific, high affinity binding sites on GHRH-R was demonstrated in all of the seven human pediatric solid tumor samples investigated. Our results show that the expression of GHRH and SV1 of GHRH-R in hemato-oncological diseases in children can pave the way for further investigation of GHRH-Rs as potential molecular targets for diagnosis and therapy.

## 1. Introduction

**Figure 1:**
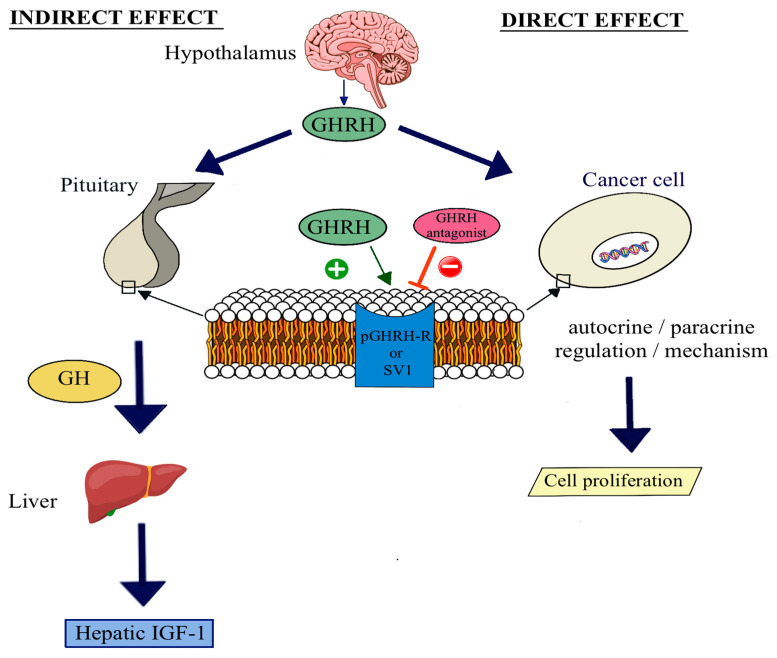
Hormonal activities of GHRH and its antagonistic analogs via indirect and direct pathways (also reviewed in detail in [5,6,7]).

Growth hormone-releasing hormone (GHRH) is a peptide neurohormone secreted by the hypothalamus. Generally, it is known to act on the pituitary gland to stimulate the production and release of growth hormone (GH) through its binding to GHRH receptors (GHRH-R) [1,2,3,4,5,6,7]. In addition to this endocrine role, the biological actions of this 44-amino acid peptide are not limited to the pituitary–hypothalamic axis, since it was demonstrated that GHRH can function as an autocrine/paracrine growth factor in various extrapituitary tissues and several human cancers [1,2,3,4,5,6,7]. The presence of the mRNA of GHRH was detected in human prostate, breast, ovarian, endometrial, pancreatic and adrenal cancers [4] and cancer cell lines derived from breast, endometrial, ovarian, prostatic, pancreatic, gastric, colorectal, lung and brain tissues, and bone sarcomas, lymphomas and renal-cell carcinomas [1,4,5,6,7,8,9,10,11]. The hormonal activities of GHRH and its analogs are mediated by the full-length pituitary type GHRH receptor (pGHRH-R) [1,3,4]. However, previous investigations have also identified splice variants (SVs) of GHRH-Rs in human cancers and other extrapituitary tissues that can mediate the effects of GHRH and its agonistic and antagonistic analogs [1,4,5,6,7,8,9,10,11] (Figure 1).

Rekasi et al. (2000) reported that the sequence of the major splice variant SV1, is almost identical to the pGHRH-R expressed in the pituitary [12]. As opposed to pGHRH-R, the first three exons were replaced by a fragment of retained intron 3 possessing a new putative in-frame start codon in SV1. This slight change can result in a partial loss of the extracellular part of the pGHRH-R protein [12]. When investigating the putative receptor protein structure of SVs, SV1 seems to be the most probable functional GHRH-R. It was also reported that SV1 binds GHRH and its antagonistic analogs with high binding affinity and mediates responses to GHRH [1,4,7,12]. Newly synthetized GHRH antagonists such as JMR-132, MZ-J-7-118, MZ-5-156, MIA-602 and MIA-690, in addition to their indirect antineoplastic actions through the GHRH-pituitary GH-hepatic insulin-like growth factor-I (IGF-I) axis, were demonstrated to directly inhibit the proliferation of experimental human cancer cell lines in vitro (Figure 1) [1,4,7,8,9]. Moreover, various studies showed that these potent GHRH antagonists with strong anticancer effects were able to inhibit the growth of several human cancer models in vivo [1,4,7,8,9,10,11]. These studies proved that the antiproliferative functions in most of these tumors were mediated by SV1 (Table 1).

Pediatric hematological and oncological diseases are major causes of pediatric mortality. In many countries, cancer is the second most common cause of death among children 1 to 14 years old, surpassed only by accidents and is the fourth most common cause of death among adolescents 15 to 19 years old [13]. Unfortunately, the overall incidence of hematological diseases and cancer in children and adolescents has increased over the past decade [14]. Based on these facts, there is an urgent need for improved diagnostic methods for early detection of these disorders and development of newer approaches to the treatment of these malignancies.

Although the expression of GHRH, GHRH-Rs and their SVs have previously been demonstrated in various human tumors and disorders [1,4,7,8,9,10,11,12], to the best of our knowledge there is no, or only very limited, existing information about the presence and gene expression of GHRH and its receptors and binding characteristics of GHRH-R SVs in pediatric hemato-oncological diseases, including various neoplastic conditions. In our study we were able to collect and investigate clinical samples of the following types of human pediatric hematological and oncological disorders: Hodgkin lymphoma (HL), rhabdomyosarcoma (RMS), teratoma (TR), acute lymphoblastic leukemia (ALL), fibrous dysplasia (FD), mesenchymal hamartoma (MH), juvenile myofibromatosis (JM), chronic benign neutropenia (CBN), hereditary spherocytosis (HS) and immune thrombocytopenia (ITP) [13,14,15,16,17,18,19,20,21,22,23,24,25,26,27,28,29,30].

The aim of our present study was to investigate the expression of mRNA for GHRH and SVs of GHRH-Rs using RT-PCR in a cohort of these human pediatric hemato-oncological disorders. In the various pediatric specimens, the protein expression and binding characteristics of GHRH-Rs were also studied by Western blot and radio ligand competition assays. To the best of our knowledge our work represents the first report demonstrating the expression of GHRH and GHRH-R SVs in various childhood tumors and other neoplastic conditions. Overall, our results will not only broaden the horizon of pediatric cancer research, but also can contribute to the ultimate goal of the development and establishment of novel potential diagnostic and therapeutic applications of new highly potent GHRH peptide analogs in pediatric oncology and hematology.

## 2. Results

### 2.1. PCR Analyses of the Expression of GHRH and SV1 of GHRH-Rs

**Figure 2:**
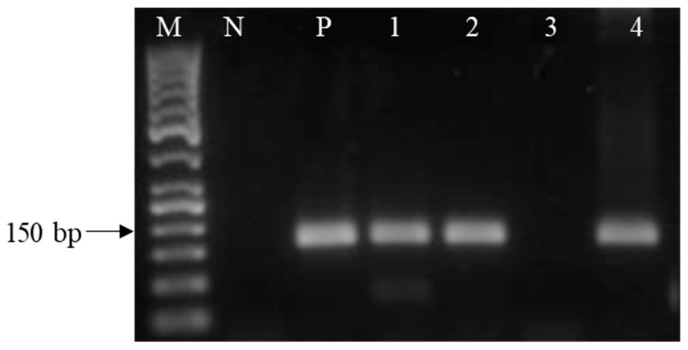
Representative RT-PCR analysis of mRNA for the GHRH ligand in human pediatric tumor specimens. The PCR products were of the expected size of 150 bp for the GHRH-L; Lane M, molecular marker (50-bp DNA ladder); Lane N, no template control; Lane P, positive control (human pituitary tissue); Lane 1: RMS, Lane 2: ALL, Lane 3: CBN, Lane 4: HL.

RT-PCR analyses revealed the presence of 150 bp products corresponding to the GHRH peptide ligand in the pediatric specimens investigated (Figure 2). Of the 15 specimens studied, eleven pediatric samples (73%) showed the expression of mRNA for GHRH (Table 2). CBN, HS, ITP and only one of the ALL specimens did not express GHRH (Table 2).

**Figure 3:**
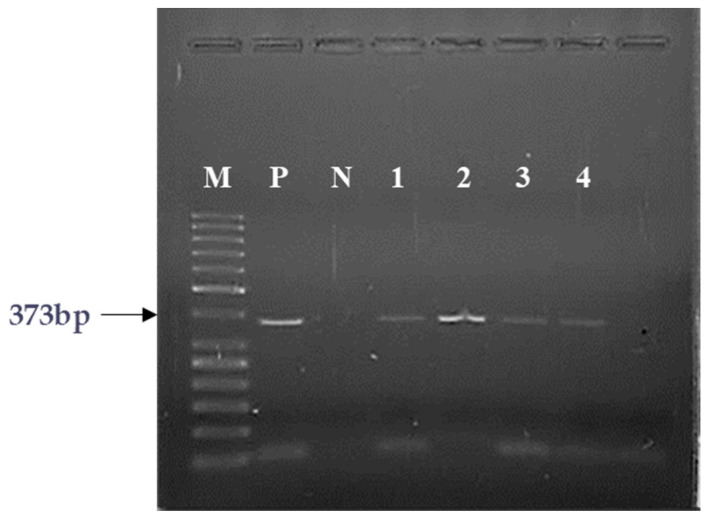
Representative RT-PCR analysis of mRNA for SV1 of GHRH-R in human pediatric tumor specimens. The PCR products were of the expected size of 373 bp for the SV1 isoform of GHRH-R; Lane M, molecular marker (50-bp DNA ladder); Lane P, positive control (human pituitary tissue); Lane N, no template control; Lanes 1: RMS, Lane 2: TR, Lane 3: HL, Lane 4: FD.

To investigate whether receptor protein and specific, high affinity binding sites for GHRH present in the membrane fractions of human pediatric hematological and oncological specimens are the products of mRNA expression of GHRH-Rs, we performed RT-PCR analyses. Reverse transcription of RNA, followed by PCR amplification with specific primers amplified 373 bp PCR products (Figure 3) which corresponded to SV1 of the GHRH-R described before [7]. Similar to the GHRH mRNA studies, of the 15 specimens studied eleven pediatric samples (73%) showed the expression of mRNA for SV1 (Table 2). CBN, HS, ITP and only one of the ALL specimens did not express mRNA for SV1 (Table 2). Negative controls yielded no detectable signals, indicating that PCR products were generated from cDNA and not from genomic DNA or other contaminants.

### 2.2. Western Blot Analysis

**Figure 4:**
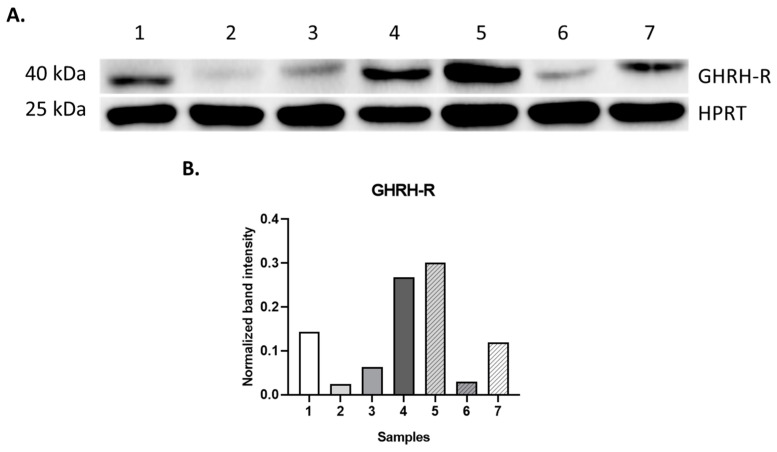
Western blot analysis of GHRH-receptor protein in human pediatric hematological/oncological specimens. Protein lysates obtained from the samples were separated on 10% SDS-PAGE (**A**). The size of the proteins was defined using a molecular weight marker (Precision Plus Dual Color Protein Standard, Bio-Rad). HPRT (hypoxanthine phosphoribosyl transferase) protein was detected as a loading control to quantify the level of GHRH-R protein in each sample using Bio-Rad Image Lab 5.2.1 software (**B**). Lanes: 1: HL, 2 and 3: RMS, 4: TR, 5: BMH, 6: FD, 7: JM.

According to Western blot analysis, the GHRH-R protein was found to be expressed in all of the seven human pediatric solid tumor samples examined (Figure 4, Table 3). In our Western blot analyses using anti-GHRH-R antibody (GHRH-R polyclonal antibody: PA3-117, Life technology, Carlsbad, CA, USA) major bands at a molecular weight of 40 kDa were demonstrated, which, according to the literature, correspond to SV1 of GHRH-R in the examined pediatric samples (Figure 4) [7]. Remarkably, some of the samples examined showed high expression of the receptor protein, and in a few of the examined specimens the protein level of GHRH-R was detected with a weaker signal on the blot.

### 2.3. GHRH Receptor Binding Studies

The presence of specific GHRH-R binding sites and the characteristics of specific binding of ^125^I-labeled GHRH antagonist JV-1-42 to membrane receptors on pediatric solid tumor samples were investigated using radioligand competition assays. We were able to examine 7 samples and our study showed that all specimens investigated, including two benign tumor samples and five malignant tumors, showed GHRH-R binding (Table 3). Analyses of the displacement studies and the Scatchard plots revealed that a one-site model provided the best curve fitting, indicating the expression of one single class of high-affinity GHRH receptor in cell membranes of human pediatric specimens. The computerized analyses of the GHRH-R binding points in the seven pediatric samples showed that the single class of GHRH-Rs had a mean dissociation constant (Kd) of 4.57 nM (range, 1.35–8.99 nM). The mean receptor concentration of GHRH-Rs (Bmax, maximal binding capacity) was 375.7 fmol/mg membrane protein (range, 222.1–733.0 fmol/mg membrane protein) (Table 3). Based on our radiolabeled receptor studies, the binding of GHRH analog [^125^I]JV-1-42 was specific, time- and temperature-dependent and reversible, demonstrating basic biochemical parameters and specifications to establish the identity of receptor binding in the human specimens investigated. The results of the ligand competition assays correlated well with the Western blot findings, demonstrating that the presence of GHRH-R protein was 100% consistent with the expression of specific binding sites of ^125^I-labeled GHRH analog JV-1-42 (Table 3).

Our results also showed that the receptor protein findings by Western blot and ligand binding assays were accompanied by the expression of mRNA for the SV1 subtype of GHRH-Rs in all pediatric specimens examined. Based on the comparative analysis of the results of the GHRH-R protein analyses and the SV1 subtype mRNA studies, we found a strong correlation investigating seven specimens. These findings showed that the expression of mRNA for the SV1 subtype was 100% consistent with the presence of GHRH-R proteins studied by Western blot and specific, high affinity binding of the GHRH antagonist [^125^I]JV-1-42 investigated by radioreceptor assays.

## 3. Discussion

In the past two decades, the function, potential role and expression of GHRH and its tumoral receptors were already investigated in various human cancers [1,4,7,8,9,10,11,12]. However, published data and information on GHRH and SVs of GHRH-Rs in pediatric cancers are very limited. Here we studied mRNA levels for GHRH and SVs of GHRH-Rs using RT-PCR, the manifestation of the GHRH-R protein by Western blot and binding characteristics of GHRH-R SV1 using a radiolabeled receptor assay in a cohort of human pediatric neoplastic conditions including Hodgkin lymphoma (HL), rhabdomyosarcoma (RMS), teratoma (TR), acute lymphoblastic leukemia (ALL), fibrous dysplasia (FD), benign mesenchymal hamartoma (BMH), juvenile myofibromatosis (JM), chronic benign neutropenia (CBN), hereditary spherocytosis (HS) and immune thrombocytopenia (ITP) [15].

In HL, intralesional non-malignant mononuclear and polymorphonuclear leukocytes outnumber pathologic Hodgkin-Reed-Sternberg cells of B-lineage origin. The 5 year-survival rates of pediatric cases exceed 95% [13,14,15,31]. In fact, pediatric HL is the first and only neoplastic single entity in Hungary, with a 5 year-survival rate of 100% according to the most recent data of the Hungarian National Childhood Cancer Registry [32]. RMS represents the most frequent form (~19%) of soft tissue sarcomas in the pediatric population with a 5–8% relative incidence in childhood cancers. RMS occurs mostly in children and young adults (<45 years-of-age), with a slight male dominance, and it is found more frequently in patients belonging to Caucasian race [15]. RMS is chemo- and radiosensitive; however, molecular lesions identified so far offer a very limited number for actionable therapeutic agents [32].

TR are solid germ cell tumors (GCT). They can present in gonadal and extragonadal locations, the latter arising in the midline either extracranially (e.g., in sacral and coccygeal bones, and mediastinum) or intracranially (e.g., certain corpus pineal tumors). Mature teratomas are usually benign, however, immature GCTs can have malignant components characterized by a fast spread that does not recognize the border of organs and may form metastatic lesions. Teratomas are formed by different types of tissues, with an incidence of ~1:4000 births [17]. ALL is the most frequent form of pediatric cancer, with a relative incidence of ~25% below the age of 15 years. The transformed cells belong to the lymphoid lineage, infiltrate bone marrow and may infiltrate various extramedullary organs, such as spleen, liver, lymph nodes, skin, bones, ovaries, testicles, and the central nervous system. The 5-year survival rate is approaching 90% among children (<15 years-of-age) and is exceeding 75% among adolescents (between 15 and 18 years of age) [18,19,20,32]. FD is a congenital, usually sporadic, benign disease, characterized by disruption of physiological bone formation where foci of osteoblasts forming a mineralized environment are embedded in a fibrotic matrix. FD can affect a single bone or multiple bones, including cranial bones. The peak incidence of FD is between 3 and 15 years [21,22]. BMH is a benign tumor of childhood and usually arises from mesenchymal tissue components. Clinical manifestation of MH is exceptional after adolescence [23]. The first clinical manifestation signs of JM usually present in infancy. Histologically, the lesion can be defined as a solitary or multiple benign mesenchymal mass involving, most frequently, soft tissues of the trunk and extremities. In addition, the head-and-neck region and visceral organs can also be affected. JM can exhibit and take an aggressive course, resulting in the death of the patient [24,25,26,27]. Granulocytopenia (GP) represents a heterogeneous group of disorders characterized by a low absolute neutrophilic polymorphonuclear cell count (ANC < 0.5 G/L). Either bone marrow failure or enhanced clearance from the circulatory pool can result in GP (28–30). CBN is a frequent condition among these patients. Mild-to-moderate isolated neutropenia is the only abnormal finding present in the complete blood count. Patients rarely develop severe infections and usually outgrow their disease by 3–5 years of age. Leukemic transformation in CBN is exceptional [13,28,29]. HS is the most common congenital hemolytic anemia north of the subtropical and Mediterranean regions. Increased red cell destruction is a result of disorders in cell membrane-associated proteins of the cytoskeleton [13,15]. In ITP, formation of autoantibodies against platelet antigens increases the clearance of platelets from the circulation, resulting in isolated thrombocytopenia. The spontaneous cure rate is high (~80% in children and 50~ in adults), and malignant transformation does not occur. However, patients often require treatment to decrease morbidity of thrombocytopenic bleeding events and to improve quality of life [30].

Of the 15 human specimens studied in our experiments, eleven pediatric samples (73%) showed the expression of mRNA for GHRH. These 11 samples also expressed mRNA for GHRH receptor SV1. GHRH-R protein was found to be expressed in two benign tumor samples and five malignant tumors examined by Western blot. The presence of specific, high affinity binding sites on GHRH-R was demonstrated in all of the seven human pediatric solid tumor samples investigated. This is in agreement with the recent report of Jimenez et al. [33] who demonstrated the presence of GHRH-R in three human acute myeloid leukemia (AML) cell lines KG-1a, K-562 and THP-1 and in nine specimens from patients with AML. Significant inhibition of cell proliferation in these cell lines following treatment with GHRH antagonist MIA-602 was found in vitro. In addition, treatment with MIA-602 of mice bearing tumor xenografts of these three human AML models, resulted in effective tumor growth inhibition [33]. To the best of our knowledge, this is the only publication about the expression of GHRH-R and a potential role of GHRH-R signaling in the pathophysiology of a neoplastic hematological disorder such as AML. In this context, it is worth mentioning that the investigated single sample of pediatric HL and 4/5 samples of childhood ALL, expressed both GHRH and SV1 in contrast to samples of CBN, HS, and ITP, which contain non-transformed blood and bone marrow cells.

Our results, showing a marked incidence of GHRH and SV1 of GHRH-R in neoplastic hematological and oncological disorders in children, support the merit of further investigation of GHRH-Rs as potential molecular targets for diagnosis and therapy. However, we are aware that a limitation of our study is the small sample size. This problem is particularly prominent if we consider the number of different subsets of samples studied. However, despite the limited number of investigated samples we were able, for the first time, to demonstrate the presence of GHRH and GHRH-Rs in neoplastic lesions of children, both at the RNA and protein levels. Although the size of the investigated cohort is rather small and its composition is heterogenous, it is worth noting that the examined specimens displayed marked expression of GHRH and GHRH-R SV1. Therefore, in the near future we would like to extend our investigation and we are trying to collect a reasonable number of samples. Hopefully, based on the findings in this pilot study investigating 15 specimens, additional human specimens from children will be able to clarify our further questions and can provide novel data about the potential clinical significance of GHRH and GHRH-Rs for diagnostic and therapeutic applications in children.

These new findings may offer a novel innovative therapeutic approach for these malignancies based on potent GHRH receptor antagonists and suggest a possible function of GHRH-R signaling in the pathology of various pediatric onco-hematological and proliferative disorders. This proposal appears to be supported by our present results that in the case of non-proliferative pediatric disorders (HS, CBN, ITP), neither GHRH mRNA nor mRNA for SV1 could be detected.

## 4. Materials and Methods

### 4.1. Sample Collecting

Specimens of pediatric hematological and oncological diseases were obtained from 15 children. Clinicopathological data, including type of tumor or clinical condition based on histopathologic features, sample origin of clinical specimens, sex, age at diagnosis and survival of the pediatric patients are shown in Table 4. The average age of children was 8.03 years (range: 9 months–15 years). Human specimens were collected from children treated at the Department of Pediatric Hematology-Oncology, University of Debrecen, Hungary. In our study, solid tumor samples were investigated from seven children, and another eight patients had malignant or benign types of hematological disorders. Seven specimens represented bone marrow aspirates, and one hematological sample was collected from peripheral blood (Table 4). Solid tumor tissues were always obtained at the time of primary surgery. Each and every sample was processed for routine histopathological examination. The final pathological diagnosis was always confirmed by an expert pathologist. Our research work was conducted in accordance with the Declaration of Helsinki, the collection and use of these pediatric samples for the current study was approved by the University of Debrecen Local Institutional Ethics Committee (DERKEB/IKEB 2284-004, date approved 4 December 2014) and informed consent was obtained.

For molecular biology and receptor protein analyses, human specimens from children were immediately frozen in liquid nitrogen, then, all samples were stored at −80 °C until in vitro studies. All diagnostic interventions were performed based on suspected neoplastic conditions.

### 4.2. RNA Isolation and Reverse Transcription Reaction (RT-PCR)

Total RNA isolation was performed from each tested pediatric sample and from a pool of human pituitaries by following the protocol of the RNA isolation kit (740933.50, Macherey-Nagel, Düren, Germany).

A NanoDrop ND-1000 spectrophotometer (Nanodrop Technologies, Wilmington, DE, USA) was used to determine the concentration and purity of the RNA. RNA samples of 1.8–2.0 optical density at 260/230 nm were only used for RT-PCR reaction and further gene expression analyses. Until further molecular biology analyses, the RNA samples were stored at −80 °C.

### 4.3. Reverse Transcription PCR (RT-PCR)

A total of 250 ng of isolated RNA from each tissue sample was reverse transcribed into cDNA in the C 1000 Touch Thermal Cycler PCR system (Bio-Rad Laboratories, Irvine, CA, USA) using a Tetro cDNA Synthesis Kit (BIO-65043, Bioline, London, UK).

Experiments were conducted in accordance with the manufacturer’s guidelines. The RT-PCR reaction was performed in a 20 μL final volume using random hexamers. The reaction for RT-PCR for one sample consisted of the following components (primer: random hexamer (1 µL), 10 mM of the dNTP mix (1 µL), 5× RT buffer (4 µL), RiboSafe RNase Inhibitor (1 µL), Tetro Reverse Transcriptase (all reagents from Bioline, London, UK), (1 µL), DEPC-treated water (20 − (n + 8)) µL, where n (for each sample is different) is the amount of total RNA used in the reaction which was calculated based on the measured RNA concentration of the samples.

The run consisted of 35 cycles (95 °C for 15 s, 60 °C for 30 s, 72 °C for 10 s, and 72 °C for two minutes). To test for contamination, RT-NTC was incorporated into the reaction.

### 4.4. RT-PCR Reaction for GHRH and SV1 of GHRH-R

The RT-PCR reaction for the detection of GHRH and SV1 in pediatric samples was performed in a 25 μL final reaction volume with gene specific primers. The primer sequences for GHRH and SV1 are found in Table 5. Normal human pituitary tissue was used as a positive control, and β-actin (ACTB) was used as a housekeeping gene. Negative samples were run for testing the clarity of the RT-PCR reaction. The RT-PCR reaction consisted of 35 cycles (95 °C for 15 s, 60–67 °C Tm annealing for 30 s, 72 °C for 10 s) and the last extension at 72 °C was for 2 min. PCR products were separated in a 1.5% agarose gel containing GelRed (G-725-100,Bioline, London, UK) and detected with UV light and digitalized with AlphaDigiDoc™ RT (Alpha Innotech, Santa Clara, CA, USA). To determine the size of the DNA, 25 or 50 bp DNA marker (BIO-33054, Bioline, London, UK) was used.

### 4.5. Western Blot Analysis of GHRH-R Protein

Pediatric tissue samples were homogenized and lysed in ice-cold protein lysis buffer (M-PER, Thermo Fisher Scientific, Waltham, MA, USA), which was supplemented with protease and phosphatase inhibitors (Sigma-Aldrich, St. Louis, MO, USA). The Bradford assay was applied for protein quantification. Before Western blotting, proteins were diluted with 4× Laemmli buffer (1610747, Bio-Rad Laboratories, Irvine, CA, USA) and 40 μg of each protein sample in equal volumes were denatured at 95 °C for 8 min. Afterwards, protein lysates were separated on a 10% sodium dodecyl sulfate-polyacrylamide gel by electrophoresis (SDS-PAGE). To define the size of the separated proteins, using Precision Plus Dual Color Protein Standard as molecular weight marker (1610374, Bio-Rad Laboratories, Irvine, CA, USA) was also loaded on the SDS-PAGE. Then, proteins were transferred to a polyvinylidene fluoride (PVDF) membrane (Millipore, Burlington, MA, USA). Non-specific binding sites were blocked with 5% milk-TBS-Tween, followed by the incubation of the membranes with the following primary antibodies overnight, at 4 °C: anti-GHRH-R antibody (1:1000 dilution, GHRH-R polyclonal antibody: PA3-117, Thermo Fisher Scientific, Waltham, MA, USA) and anti-HPRT antibody (1:1000 dilution, P00492 rabbit monoclonal; Biol. Technology, Carlsbad, CA, USA). HPRT as a housekeeping protein was measured to ensure equal loading of all samples. After extensive washing steps with TBS-Tween, membranes were incubated with anti-rabbit IgG secondary antibody (1:5000, Thermo Fisher Scientific, Waltham, MA, USA—diluted in 5% skin milk in TBS-Tween) for two hours at room temperature. Then, after washing membranes with TBS-Tween several times, the signal was detected by chemiluminescence using Clarity Western ECL Substrate (Bio-Rad Laboratories, Hercules, CA, USA). The intensity of each band was normalized to HPRT. Quantification was carried out using the Bio-Rad Image Lab 5.2.1 software (Bio-Rad Laboratories, Hercules, CA, USA).

### 4.6. Preparation of Membranes and Radioligand Binding Studies

For the radioreceptor binding studies, GHRH antagonist JV-1-42 was radioiodinated by the chloramine-T method, as previously reported, and then purified by HPLC [6,10,12]. Cell membrane preparation from human pediatric solid tumor specimens for the receptor binding studies using radioligand competition assays was carried out as described earlier [6,10,12]. Protein lysate preparation from the pediatric tissue samples was performed according to the next steps: the pediatric solid tumor samples were first defrosted and then homogenized using a tissue homogenizer (Ultra-Turrax, IKA Works, Wilmington, NC, USA) in 50 mmol/L Tris-HCl buffer (pH 7.4), supplemented with protease inhibitors (0.25 mmol/L PMSF: phenylmethylsulfonylfluoride, 2 μg/mL pepstatin A, and 0.4% aprotinin) (Merck, Darmstadt, Germany). The final crude membrane fractions were prepared and stored at −80 °C until further in vitro investigations, as described earlier in the literature [6,10,12]. The amount of protein was determined by the Bradford assay. GHRH-R binding investigations were accomplished, as reported in our protocols previously [6,10,12]. In vitro ligand competition assays were performed based on the binding of [^125^I]JV-1-42 as radioligand to membrane fractions of human pediatric samples. High affinity binding properties of the radioligand [^125^I]JV-1-42 GHRH antagonist to rat and human pituitaries were reported earlier [4,6,10,11,12]. This GHRH antagonist also has a high binding affinity to various human cancers, such as renal, endometrial, prostate and breast [4,6,10,11,12]. The specific, high affinity binding of radiolabeled JV-1-42 to SV1 was also demonstrated and reported in the literature [1]. In detail, membrane homogenates were incubated with radioligand [^125^I]JV-1-42 and increasing concentrations (10^−12^–10^−6^ mol/L) of non-radioactive competitor peptides in binding buffer. After a one hour incubation and separation, the receptor bound fraction in the final pellet was counted in a γ-counter. A curve-fitting computer program (LIGAND-PC) of Munson and Rodbard was used to calculate the binding characteristics (Kd and Bmax) of GHRH-Rs. Due to the very small sample size and limited amounts of membrane fractions, the GHRH ligand competition studies were performed in only seven specimens.

## 5. Conclusions

Our findings pave the way for further development of novel, highly potent GHRH peptide analogs for GHRH-R based targeted tumor therapy, as valid alternative options to the current treatment strategies [1,4,7,15,34,35,36].

## Figures and Tables

**Table 1:** Experimental cancer models and neoplastic hematological disorders where the antiproliferative effects of various GHRH antagonists were demonstrated.

Models Investigated	
Breast	Renal
Ovarian	Osteosarcoma
Endometrial	Ewing sarcoma
Prostate	Glioblastoma
Lung	Esophageal squamous cell carcinoma
Pancreatic	Pleural mesothelioma
Colorectal	Acute myeloid leukemia

Reviewed in references [1,4,7,8,9,10,11,12].

**Table 2:** Expression of mRNA for GHRH and SV1 in pediatric samples.

Sample	Clinical Condition	GHRH mRNA	SV1 mRNA
1	HL	+	+
2	RMS	+	+
3	RMS	+	+
4	TR/adenocarcinoma components	+	+
5	ALL	+	+
6	ALL	+	+
7	ALL	+	+
8	ALL	−	-
9	ALL	+	+
10	BMH	+	+
11	FD	+	+
12	JM	+	+
13	CBN	−	−
14	HS	−	−
15	ITP	−	−

HL: Hodgkin lymphoma, RMS: rhabdomyosarcoma, TR: teratoma with adenocarcinoma components, ALL: acute lymphoblastic leukemia, BMH: benign mesenchymal hamartoma, FD: fibrous dysplasia, JM: juvenile myofibromatosis, CBN: chronic benign neutropenia, HS: hereditary spherocytosis, ITP: immune thrombocytopenia.

**Table 3:** Presence and binding characteristics of GHRH-R protein in pediatric samples.

Sample	Clinical Condition	GHRH-R Protein	GHRH-R Binding	
			Kd (nM)	Bmax (fmol/mg Prot.)
1	HL	+	1.37	398.7
2	RMS	+	3.84	222.1
3	RMS	+	4.38	245.0
4	TR/adenocarcinoma components	+	8.99	475.0
5	ALL	N/A	N/A	
6	ALL	N/A	N/A	
7	ALL	N/A	N/A	
8	ALL	N/A	N/A	
9	ALL	N/A	N/A	
10	BMH	+	7.27	733.0
11	FD	+	4.81	261.4
12	JM	+	1.35	294.6
13	CBN	N/A	N/A	
14	HS	N/A	N/A	
15	ITP	N/A	N/A	

HL: Hodgkin lymphoma, RMS: rhabdomyosarcoma, TR: teratoma with adenocarcinoma components, ALL: acute lymphoblastic leukemia, BMH: benign mesenchymal hamartoma, FD: fibrous dysplasia, JM: juvenile myofibromatosis, CBN: chronic benign neutropenia, HS: hereditary spherocytosis, ITP: immune thrombocytopenia, N/A: not applied.

**Table 4:** Clinicopathology data of fifteen pediatric hemato-oncological samples.

Sample	Clinical Condition	Sex	Sample Origin	Age at Sampling	Disease Outcome
1	HL	F	Lymph node	15 years	Alive
2	RMS	M	Pelvic tumor tissue	12.5 years	† Died
3	RMS	F	Neck mass biopsy	8 years	† Died
4	TR/adenocarcinoma components	M	Mediastinal tumor tissue	14.5 years	† Died
5	ALL	F	Bone marrow	5 years	Alive
6	ALL	M	Bone marrow	13 years	Alive
7	ALL	M	Bone marrow	9.5 years	† Died
8	ALL	M	Bone marrow	6.5 years	Alive
9	ALL	M	Peripheral blood	15 years	† Died
10	BMH	M	Mediastinal mass	4 years	Alive
11	FD	F	Bone tumor tissue	10.5 years	Alive
12	JM	M	Right pelvic bone tumor tissue	2 years	Alive
13	CBN	M	Bone marrow	12.5 months	Alive
14	HS	F	Bone marrow	9 months	Alive
15	ITP	M	Bone marrow	10 months	Alive

HL: Hodgkin lymphoma, RMS: rhabdomyosarcoma, TR: teratoma with adenocarcinoma components, ALL: acute lymphoblastic leukemia, BMH: benign mesenchymal hamartoma, FD: fibrous dysplasia, JM: juvenile myofibromatosis, CBN: chronic benign neutropenia, HS: hereditary spherocytosis, ITP: immune thrombocytopenia; F: female, M: male.

**Table 5:** Sequences of ACTB, GHRH and SV1 primers and other parameters used for the RT-PCR assay.

Primers	Forward Sequences	Reverse Sequences	Tm (°C) Annealing Temperature	Product Size (bp)
ACTB	5′-GGCATCCTCACCCCCTGAAGTA-3′	5′-GGGGTGAAGGTCTCAAA-3′	60 °C	203 bp
GHRH	5′-GGAGTTGTGGCTAGAGAG-3′	5′-TGTCTGTCTACCTGACGACCAA-3′	64 °C	150 bp
SV1	5′-GGAGTTGTGGCTAGAGAG-3′	5′-GCATAGAACAGTGGAGAAG-3′	67 °C	373 bp

## Data Availability

The data presented in this study are available on request from the corresponding authors.
